# The mTOR pathway controls phosphorylation of BRAF at T401

**DOI:** 10.1186/s12964-024-01808-2

**Published:** 2024-09-02

**Authors:** Daniel Christen, Manuel Lauinger, Melanie Brunner, Jörn Dengjel, Tilman Brummer

**Affiliations:** 1 Institute of Molecular Medicine, University of Freiburg, Stefan-Meier-Str. 17, 79104 Freiburg, Germany; 2 Faculty of Biology, University of Freiburg, Freiburg, Germany; 3 German Cancer Research Center (DKFZ), German Cancer Consortium (DKTK), Partner Site Freiburg and, Heidelberg, 69120 Germany; 4 Department of Biology, University of Fribourg, Chemin du Museé 10, 1700 Fribourg, Switzerland; 5 Comprehensive Cancer Center Freiburg (CCCF), Medical Center, Faculty of Medicine, University of Freiburg, University of Freiburg, 79106 Freiburg, Germany; 6 Center for Biological Signalling Studies BIOSS, University of Freiburg, 79104 Freiburg, Germany

## Abstract

BRAF serves as a gatekeeper of the RAS/RAF/MEK/ERK pathway, which plays a crucial role in homeostasis. Since aberrant signalling of this axis contributes to cancer and other diseases, it is tightly regulated by crosstalk with the PI3K/AKT/mTOR pathway and ERK mediated feedback loops. For example, ERK limits BRAF signalling through phosphorylation of multiple residues. One of these, T401, is widely considered as an ERK substrate following acute pathway activation by growth factors. Here, we demonstrate that prominent T401 phosphorylation (pT401) of endogenous BRAF is already observed in the absence of acute stimulation in various cell lines of murine and human origin. Importantly, the BRAF/RAF1 inhibitor naporafenib, the MEK inhibitor trametinib and the ERK inhibitor ulixertinib failed to reduce pT401 levels in these settings, supporting an alternative ERK-independent pathway to T401 phosphorylation. In contrast, the mTOR inhibitor torin1 and the dual-specific PI3K/mTOR inhibitor dactolisib significantly suppressed pT401 levels in all investigated cell types, in both a time and concentration dependent manner. Conversely, genetic mTOR pathway activation by oncogenic RHEB (Q64L) and mTOR (S2215Y and R2505P) mutants substantially increased pT401, an effect that was reverted by dactolisib and torin1 but not by trametinib. We also show that shRNAmir mediated depletion of the mTORC1 complex subunit Raptor significantly enhanced the suppression of T401 phosphorylation by a low torin1 dose, while knockdown of the mTORC2 complex subunit Rictor was less effective. Using mass spectrometry, we provide further evidence that torin1 suppresses the phosphorylation of T401, S405 and S409 but not of other important regulatory phosphorylation sites such as S446, S729 and S750. In summary, our data identify the mTOR axis and its inhibitors of (pre)clinical relevance as novel modulators of BRAF phosphorylation at T401.

**Supplementary Information:**

The online version contains supplementary material available at 10.1186/s12964-024-01808-2.

## Background

Upon RAS-dependent recruitment to the plasma membrane, BRAF activates the MEK/ERK pathway [1]. Activated ERK phosphorylates thousands of proteins in the cytoplasm and nucleus, thereby controlling numerous processes in normal and malignant cells [2]. BRAF alterations are frequently observed in cancer, either as point mutants like BRAF^V600E^ or as fusion proteins resulting from chromosomal recombination [3, 4].

Under physiological conditions, BRAF is tightly regulated by a complex activation cycle, including dimerization, binding of 14-3-3 proteins and multiple feed-forward and feedback phosphorylation events. ERK-mediated phosphorylation of BRAF at S151, T401, S750 and T753, represents a rapid negative feedback loop, disrupting critical protein–protein interactions, including active RAF dimers [5–7]. Additionally, the RAS/BRAF/MEK/ERK axis is modulated by crosstalk with other pathways. For example, AKT phosphorylates BRAF at the 14-3-3 binding site S365, thereby stabilising its auto-inhibited conformation [8, 9]. The PI3K/AKT/mTOR pathway senses growth factors, nutrients, and cellular energy levels and is activated by numerous signals. Receptor tyrosine kinases (RTKs), e.g., activate phosphatidylinositol 3-kinase (PI3K) generates phosphatidylinositol-3,4,5-triphosphate (PIP_3_). PIP_3_ recruits PDK1 and mTORC2 to the plasma membrane [10] and both activate AKT/PKB by phosphorylating T308 and S473, respectively. AKT phosphorylates and inhibits the tuberous sclerosis complex (TSC), serving as an GTPase activating protein for RHEB, which in turn activates mTORC1. While both mTOR complexes share common components such as mLST8 and the catalytically active kinase mammalian target of rapamycin (mTOR), they differ in their composition, with mTORC1 binding Raptor and mTORC2 containing Rictor and mSIN1 [11]. Both complexes phosphorylate distinct substrates, with mTORC1 and mTORC2 phosphorylating S6 kinase and AKT at S473, respectively [11].

BRAF contains three conserved regions (CR), CR1 involved in RAS binding, CR2 harbouring the 14-3-3 binding site S365 and CR3 encompassing the kinase domain. Between CR2 and CR3 lies a presumed unstructured hinge region in which we identified a phosphorylation cluster around T401 [12]. We demonstrated that multiple phosphorylation sites flanking T401 contribute to the pronounced electrophoretic mobility shift of BRAF observed upon treatment with the RAF inhibitor (RAFi) sorafenib or upon introducing the kinase-impairing D594A mutation. Both conditions promote dimerization, a prerequisite for the hyperphosphorylation of most sites within the T401 cluster. Notably, T401 itself is phosphorylated even in untreated cells, suggesting that this post-translational modification (PTM) precedes cluster phosphorylation [12]. So far, T401 has been described as an ERK phosphorylation site and implicated in the feedback mediated disruption of BRAF/RAF1 heterodimers [5]. Moreover, phosphorylation of T401 and S405 generates an evolutionary conserved phospho-degron, targeting BRAF for proteasomal degradation [13–16]. These findings suggest a model in which ERK-mediated phosphorylation of T401, coupled with dimerization-induced hyperphosphorylation of adjacent residues, promotes BRAF dimer disruption and/or degradation. However, our previous observation that T401 phosphorylation (pT401) was notably present in cells with low ERK activity [12] suggests that kinases other than ERK could be involved in this process. Indeed, based on a serendipitous discovery, we now provide several lines of evidence that pT401 is mediated by the mTOR pathway, while blockade of the MEK/ERK axis shows no discernible effect.

## Methods

### Cell lines

HEK293T cells were provided in-house by Andreas Hecht. Simian Virus 40 large T antigen immortalised *Braf*^floxE12/floxE12^ murine embryonic fibroblasts (MEF) were transduced with pBABE-puro-CreERT2 for conditional *Braf* deletion, either singly or in combination with an expression cassette for the ER^T2^-HRAS^G12V^ fusion protein, were generated in-house and described in detail previously [17]. *Tsc1*^−/−^ MEFs [18] were kindly provided by Prof. Ian Frew (University Medical Centre Freiburg). All MEFs as well as HEK293T cells were cultivated in DMEM medium (4.5 g/l glucose) supplemented with 10% fetal calf serum (FCS), 2 mM L-glutamine, 10 mM HEPES, 200 U/ml penicillin, 200 μg/ml streptomycin). Transient transfection of HEK293T cells and retroviral infection of MEFs was carried out as described previously [17]. For Western blot analysis, cells were lysed with normal lysis buffer 48 h post transfection.

MCF-10A cells expressing the ecotropic retroviral receptor (MCF-10A/EcoR cells; a kind gift of Drs. Danielle Lynch and Joan Brugge) were cultivated as described previously [12]. WM3928 cells were purchased from Rockland Immunochemicals, Inc. (Rockland), Pennsylvania, USA. WM3928 were cultivated in tumor specialized Medium (80% MCDB153, 20% Leibovitz’s L-15, supplemented with 10% FCS, 2 mM L-glutamine, 200 U/ml penicillin, 200 μg/ml streptomycin, 5 µg/mL h-Insulin and 1.68 mM CaCl_2_.

### Generation of inducible RPTOR and RICTOR knock-down HEK293T cell lines

TRIPZ Inducible Lentiviral shRNA against human Rptor (Catalogue ID: RHS4740-EG57521) and Rictor (Catalogue ID: RHS4740-EG253260) were purchased from Horizon Discovery Ltd., pTRIPZ non-silencing control from Open Biosystems. HEK293T were transiently transfected with the Trans-Lentiviral packaging System (Open Biosystems) and pTRIPZ plasmid. Forty-eight hours later cell culture supernatant was collected and filtered through a 0.2 µm filter. Virus containing supernatant was then used to infect HEK293T cells (9 mL plus 5 mL fresh growth medium). Cells were selected for 7 days with puromycin (0.5 – 2 µg/mL). For induction, 0.5 µg/mL doxycycline was used for 72 h.

### Plasmids

RHEB_OHu17242C_pcDNA3.1( +)-N-Myc was ordered from GenScript Biotech Corporation. RHEB_OHu17242C_pcDNA3.1( +)-N-Myc Q64L mutant was generated by site-directed mutagenesis using the following primers: 5’-ACAGCCGGGCtAGATGAATAT-3’ and 5’-GTCTACAAGTTGAAGATGATATTC-3’ and the NEB Q5 SDM protocol. pcDNA3 mTOR^WT^, mTOR^S2215Y^ and mTOR^R2505P^ were kindly provided by Ian Frew and described earlier [19]. The retroviral pMIG and pMIG/HA-BRAF^WT^ expression vectors have been described previously [12, 17]. The pMIG/HA-BRAF^T401A^ vector was generated by site-directed mutagenesis using the primers hBRAF T401A new.FOR 5’-TTTGTCTGCTgCCCCCCCTGC-3’ and hBRAF T401A new.REV 5’-CCTGTGGTTGATCCTCCATCAC-3’ and *Q5* polymerase (nucleotide mismatches shown in lower case).

### Antibodies and reagents

Antibodies used in this study were anti-phospho-p70 S6 Kinase (Thr389) (1A5), anti-p70 S6 Kinase (49D7), anti-phospho-p44/22 (ERK1/2) (Thr202/Tyr204), anti-p44/42 MAPK (ERK1/2), anti-MYC-Tag (9B11), anti-mTOR (7C10) (all from Cell Signaling Technologies), anti-RAF-B (F-7), anti-α-Tubulin (Santa Cruz Biotechnology) and anti-BRAF (phospho T401) [EPR2208Y] (Abcam). The specificity of the latter antibody was confirmed by its inability to recognize the BRAF^T401A^ mutant (Supplementary Figure S1 and its failure to recognize dephosphorylated BRAF [20]. Inhibitors (dactolisib, torin1, alpelisib, rapamycin, trametinib, naporafenib, temsirolimus, everolimus, tacrolimus, afatinib, XL888 and ulixertinib) were purchased from SelleckChem. All inhibitors were dissolved in DMSO.

### Western blotting

Western blotting was carried out as previously described [17]. Briefly, cells were lysed in normal lysis buffer (NLB: 50 mM Tris/HCl, pH 7.5; 1% Triton X-100; 137 mM sodium chloride; 1% glycerine; 1 mM sodium orthovanadate; 0.5 mM EDTA; 0.01 mg/ml leupeptin, 0.1 mg/ml aprotinin, 1 mM AEBSF), separated on SDS gels containing 10% polyacrylamide and transferred to PVDF membranes. Blotted proteins were visualized using horseradish peroxidase-conjugated secondary antibodies (Thermo Scientific), SuperSignal West Femto Maximum Sensitivity Substrate (Thermo Scientific) and a Fusion Solo imaging system (Vilber). Signals were quantified using FusionCapt Advance. In case lysate aliquots were run on different gels, equal loading/transfer was confirmed by gel-specific loading controls.

### Immunoprecipitations

For immunoprecipitations, HEK293T cells transiently expressing HA-BRAF proteins were grown to sub-confluency in a 10 cm dish and lysed in 1 ml IP-Lysis Buffer (25 mM Tris–HCl pH 7.4, 150 mM NaCl, 1 mM EDTA, 1 mM orthovanadate, 2.5% glycerol, 1% NP-40) 48 h post transfection. Immunoprecipitation was performed using the KingFisher Duo Prime IP washer and Pierce™ Anti-HA Magnetic Beads (Thermo Scientific™). Following resuspension in 50 µl IP-Lysis Buffer, addition of 5 × Laemmli buffer and boiling for 5 min, samples were analyzed by Western blotting.

### Mass spectrometry (MS)

MEFs expressing 4-hydroxytamoxifen inducible ER^T2^-HRAS^G12V^ were cultured for 14 days in media containing L-arginine (Arg0) and L-lysine (Lys0), L- lysine–^2^H_4_ (Lys4), and L-arginine–U-^13^C_6_ (Arg6), or L-lysine–U- ^13^C_6_ -^15^N_2_ (Lys8) and L- arginine–U-^13^C_6_ -^15^N_4_ (Arg10) to generate ‘light’, ‘medium’ and ‘heavy’ labelled cells, respectively [12]. Cells were induced for 16 h with 4-HT or ethanol as control before inhibition for 4 h with torin1(1 µM). Subsequently, cells were lysed as mentioned in section “Western Blotting” and incubated for 4 h with Anti-HA Affinity Matrix beads (Roche). The matrix was then washed with NLB at least seven times by centrifugation at 400 × g for one minute.

Beads were resuspended in IP-Lysis buffer and the three SILAC labels were combined (light, medium and heavy) followed by FASP as described previously [21]. Briefly, beads were loaded on a 10 kD cut-off filter, spun (12,000 g, 10 min), proteins were reduced with 1 mM DTT in 8 M urea in 100 mM ABC buffer for 20 min at RT, followed by alkylation using 5.5 mM IAA in 8 M urea in 100 mM ABC buffer for 20 min in the dark at RT. Urea was then replaced by ABC buffer, followed by addition of trypsin (1:100 trypsin: protein ratio) and digested overnight at 37 °C. The samples were spun down the next day, acidified to 1% TFA and lyophilized prior to phosphopeptide enrichment. Samples were resuspended in 200 μL 80% acetonitrile with 0.1% TFA in deionized water for phosphopeptide enrichment using Fe(III)-NTA cartridges (Agilent). Phosphopeptide-enriched samples were lyophilized overnight and resuspended in 20 μL 0.1% formic acid for LC–MS/MS analysis.

LC–MS/MS analyses were performed on a Exploris 480 mass spectrometer coupled to an EasyLC 1200 nanoflow HPLC (all Thermo Scientific). Peptides were separated on a fused silica HPLC column tip (I.D. 75 μm, New Objective, self-packed with ReproSil-Pur 120 C18-AQ, 1.9 μm (Dr. Maisch) to a length of 20 cm) using a gradient of A (0.1% formic acid in water) and B (0.1% formic acid in 80% acetonitrile in water). Samples were loaded with 0% B with a flow rate of 600 nL/min; peptides were separated by 5%–30% B within 85 min with a flow rate of 250 nL/min. Spray voltage was set to 2.3 kV and the ion-transfer tube temperature to 250 °C; no sheath and auxiliary gas were used. The mass spectrometer was operated in data dependent mode, after each MS scan (m/z = 370 – 1′750; resolution: 120′000, AGC target value: 300%,) a maximum of twenty MS/MS scans were performed using a normalized HCD collision energy of 30%, a target value of 50% and a resolution of 60′000.

MS raw files were analyzed using MaxQuant (version 2.0.1.0) [22] using a Uniprot full-length *Homo sapiens* database (January, 2022) and common contaminants such as keratins and enzymes used for in-gel digestion as reference. Carbamidomethyl-cysteine was set as fixed modification and protein amino-terminal acetylation, serine-, threonine- and tyrosine-phosphorylation, and oxidation of methionine were set as variable modifications. The MS/MS tolerance was set to 20 ppm and three missed cleavages were allowed using trypsin/P as enzyme specificity. Peptide, site, and protein FDR based on a forward-reverse database were set to 0.01, minimum peptide length was set to 7, the minimum score for modified peptides was 40, and minimum number of peptides for identification of proteins was set to one, which must be unique. The ‘‘match between- run’’ option was used with a time window of 0.7 min. MaxQuant results were analyzed using Perseus (version 1.6.15, [23]).

### Statistical analysis

The number of individual experiments as well as the applied statistical tests are specified in the respective figure legend. Data is presented as mean ± SD, if not stated otherwise. Statistical analyses were performed using GraphPad Prism 10 (GraphPad Inc., CA).

## Results

### mTOR inhibition reduces the phosphorylation of BRAF threonine 401

**Fig. 1:**
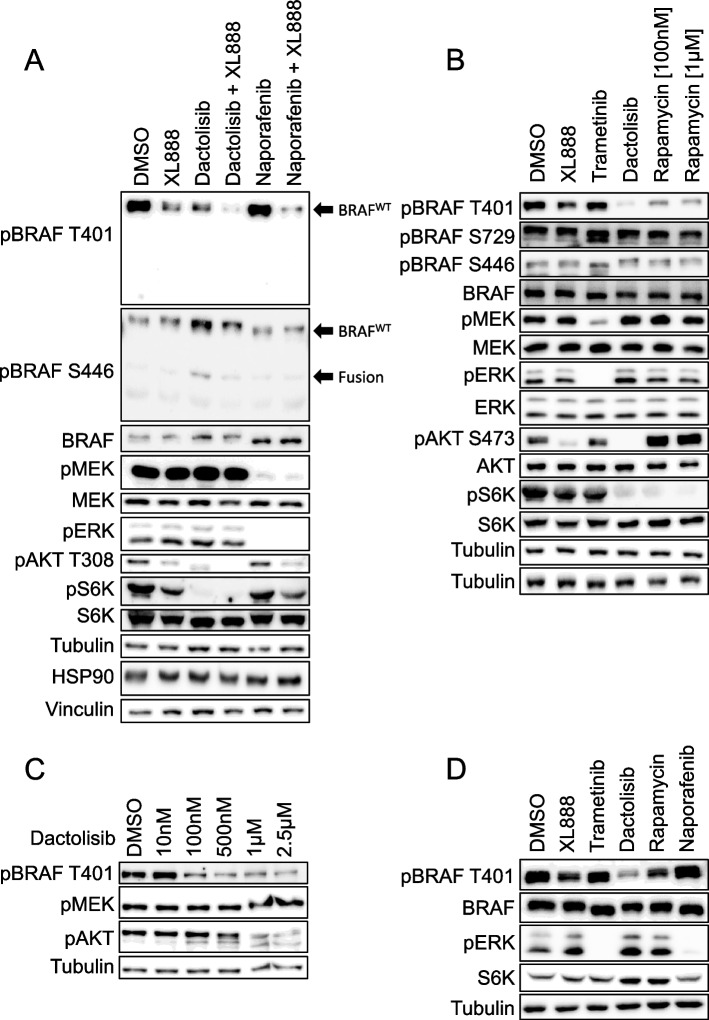
Dactolisib and rapamycin impair BRAF phosphorylation at T401. Individual Western blot analyses of WM3928 cells treated for 6 h with the indicated inhibitors variations are shown. Dactolisib was used in three experiments (**A**, **B**, and **D**) at a concentration of 2.5 µM. Naporafenib was used in (**A)** and (**B**) at 1 µM. XL888 was used at either 1 µM (**A**) or 100 nM (**B** and **D**). Trametinib was applied at a concentration of 50 nM. Rapamycin was used at 100 nM and 1 µM in (**B**) and at 100 nM in (**D**).** C** shows a titration of dactolisib. Detection of tubulin, HSP90 and vinculin, as well as total proteins, serve as loading controls

Initially, we investigated whether inhibitors of the HSP90/CDC37 chaperone complex (XL888) or the RAS/PI3K/AKT/mTOR axis modulate the ERK pathway in the melanoma cell line WM3928, which harbours the *SKAP2::BRAF* fusion oncogene [24], along with a *PTEN* loss-of-function mutation elevating AKT/mTOR signalling. Unfortunately, the detection of SKAP2::BRAF was impeded by the fact that all currently available BRAF antibodies recognize epitopes excluded from the SKAP2::BRAF fusion. To overcome this limitation, we used antibodies raised against the BRAF phosphorylation sites T401 and S446, as we knew from previous experiments [12] and literature [25] that these phosphorylation sites are readily detected (Fig. 1A). Thereby, we surprisingly observed that the dual PI3K/mTORC1/mTORC2 inhibitor dactolisib potently suppressed the phosphorylation of full-length BRAF co-expressed from the non-rearranged allele at T401 (Fig. 1A, B). As T401 is regarded as an ERK feedback phosphorylation site [5, 26], we further explored this unexpected finding with additional inhibitors. Rapamycin, a compound primarily targeting mTORC1 [27], also reduced pT401, albeit to a lesser extent. Importantly, neither dactolisib nor rapamycin affected BRAF phosphorylation status at S446 or S729, indicating their specific effect on pT401 (Fig. 1B). As expected from their target specificity, dactolisib suppressed AKT phosphorylation at the mTORC2 site S473 and of p70S6K, while rapamycin only suppressed p70S6K phosphorylation, with a concomitant increase in AKT phosphorylation at S473. This agrees with the known rapamycin-mediated disruption of multiple negative feedback loops controlling the RTK/IRS1/PI3K/AKT/mTOR axis [28]. These observations, combined with the absence of effects on MEK/ERK phosphorylation by both drugs, highlight the specific effect of mTOR inhibitors on pT401 rather than a global suppression of phosphorylation events due to toxicity. To further investigate whether the RAS/ERK pathway contributes to pT401 under different conditions, we treated WM3928 cells with the BRAF/RAF1 inhibitor naporafenib and the MEK inhibitor trametinib. Despite a profound reduction in MEK and/or ERK phosphorylation, pT401 remained unaffected by these (pre)clinically relevant compounds, while XL888, an inhibitor of the kinase-chaperoning HSP90, reduced pT401 (Fig. 1A-C).

**Fig. 2:**
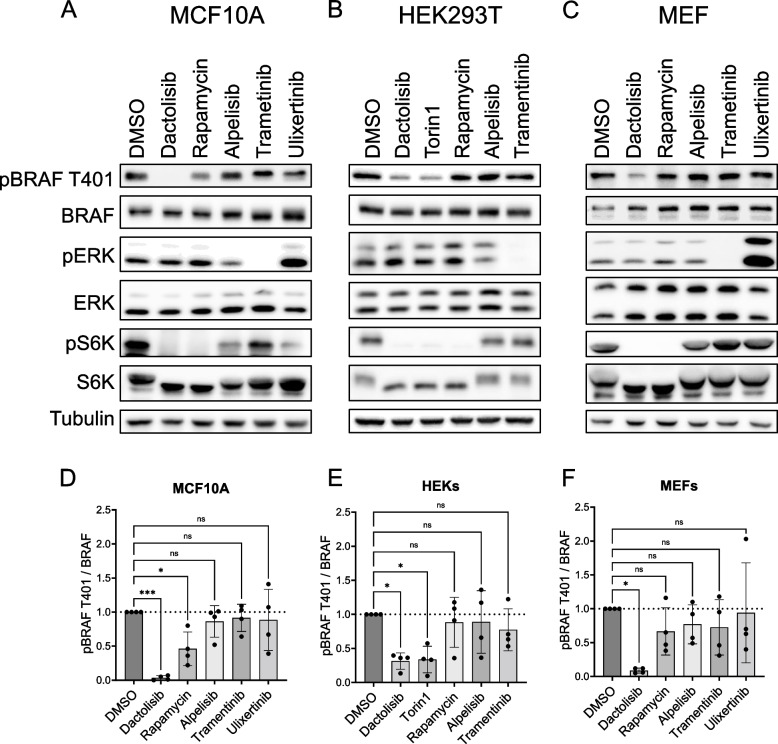
Dactolisib and torin1 impair BRAF phosphorylation at T401 across various cell types. Western Blot analysis of MCF10AecoR cells (A), HEK293T cells (**B**), and MEFs (**C**). Cells were treated with indicated inhibitors (all 1 µM, except trametinib, 50 nM) or control (DMSO) for 4 h and then subjected to Western blot analysis using the indicated antibodies. Phospho-BRAF T401 and total BRAF were quantified using FusionCapt Advance Software, normalized to α-Tubulin, and the ratio for pBRAF/BRAF was calculated for MCF10A (**D**), HEK393T (**E**), and MEF (**F**) cell lines. Ratios were normalized to DMSO. Statistical analysis: mean ± SD, *n* = 4, one-way ANOVA with Dunnett ‘s test for multiple comparisons, * *P* ≤ 0.05, ** *P* ≤ 0.01, *** *P* ≤ 0.001, **** *P* ≤ 0.0001. Detection of tubulin as well as of total ERK serves as loading control

To demonstrate that the effects of mTOR inhibitors on T401 are not restricted to WM3928, we used two additional and quite distinct human cell lines, the immortalised mammary epithelial cell line MCF10AecoR and HEK293T cells, as well as immortalised murine embryonic fibroblasts (MEFs). Consistent with our observations in WM3928 cells, dactolisib impaired pT401 in these models (Fig. 2A-C). Rapamycin (mTORC1-specific) only reduced pT401 in MCF10AecoR cells (Fig. 2A), whereas alpelisib, a specific PI3K inhibitor, caused a lesser reduction in p70S6K phosphorylation than dactolisib and did not affect the phosphorylation status of T401 across all three cell lines. Torin1, a specific mTORC1/2 inhibitor with more than 100-fold higher selectivity over PI3K family members [29], decreased pT401 in HEK293T cells to a similar extent as dactolisib (Fig. 2B, E) and in a dose-dependent manner (Supplementary Fig. S2A). This implicates mTOR complexes rather than other PI3K-dependent kinases in pT401 modulation and suggests that inhibition of the mTORC1/S6K axis is associated with loss of pT401. Interestingly, pT401 appeared more stable than p70S6K phosphorylation, in both dose–response and time-course experiments (Supplementary Fig. S2A-D). Following the wash-out of torin1, phosphorylation of p70S6K also rebounded earlier than pT401 (Supplementary Fig. S2E). These divergent kinetics of dephosphorylation and re-phosphorylation of distinct mTORC1 effector residues are reminiscent of a recent study in yeast, demonstrating that the temporally dynamic regulation of TORC1 downstream targets is in part shaped by the nature of the protein phosphatases impinging on the respective phospho-residues [30].

In addition to rapamycin, we tested the effect of other macrolides such as everolimus, temsirolimus and tacrolimus on pT401 in HEK293T cells (Supplementary Fig. 2F and G). While the directly mTORC1-inhibiting macrolides rapamycin, everolimus and temsirolimus induced, at best, a trend for reduced pT401, tacrolimus, a calcineurin inhibitor [31], significantly increased this phosphorylation event. This agrees with previous observations showing that tacrolimus may enhance mTOR activity [32] and with a study identifying pT401 as a calcineurin substrate [31]. We also assessed the specific mTORC2 inhibitor JR-AB2-011 [33], but observed no effect on pT401. However, JR-AB2-011 failed to impair AKT phosphorylation at S473 [11], questioning its efficacy to block mTORC2 in this experimental setting (Supplementary Fig. S2F). Likewise, the pan-EGFR inhibitor afatinib, trametinib, or the ERK inhibitor ulixertinib did not affect pT401 significantly, indicating the dispensability of the ERK axis for this phosphorylation event across these three cell types (Fig. 2A-F).

Using phosphoproteomics, we investigated the effect of acute RAS signalling, also in combination with torin1, on the BRAF phosphorylation status (Supplementary Fig. S3A and B). Therefore, we used *Braf*-deficient MEFs, which also harbour a 4-hydroxytamoxifen-inducible ER^T2^-HRAS^G12V^ fusion protein [12, 17], reconstituted with hemagglutinin (HA) tagged human BRAF. Consistent with our previous findings [12], this analysis revealed that activated ER^T2^-HRAS^G12V^ induces phosphorylation of anti-HA purified BRAF at the hinge region sites T401, S405, and S409, which was reverted by torin1. In contrast, torin1 did not affect other prominent phosphorylation sites such as the N-region residue S446, the C-terminal 14-3-3 binding site S729 or the ERK feedback site S750 [34], as it was observed for WM3928 cells by Western blotting (Fig. 1B). To estimate the occupancy of T401 phosphorylation, we used the non-phosphorylated and a doubly phosphorylated version of the BRAF peptide 385–414 harbouring a phosphate group at T401 and a second one which could not be clearly localized. This calculation was performed according to a published protocol [35]. Oncogenic HRAS^G12V^ increased T401 phosphorylation from 11 to 56%, whereas addition of torin1 partially blunted this response to 41% (Supplementary Figure S3C). Thus, T401 is a major phosphorylation event on BRAF and affects approximately half of the protomers. However, as we did not identify the singly phosphorylated peptide carrying only a phosphate group at T401 and as we cannot exclude the presence of more than two phosphate groups on this peptide known to contain multiple phosphorylation sites [12], these numbers should be treated with caution. Our data also highlight that the use of phospho-specific antibodies complement MS approaches and vice versa. Thus, our comprehensive analysis across four mammalian cell lines representing distinct cell lineages, demonstrate that the RAF/MEK/ERK axis is dispensable for pT401, while mTOR inhibitors, in particular dactolisib and torin1, achieve a profound reduction of this PTM.

### Genetic activation of the mTOR pathway increases BRAF phosphorylation at T401 and is reversed by mTOR inhibition

**Fig. 3:**
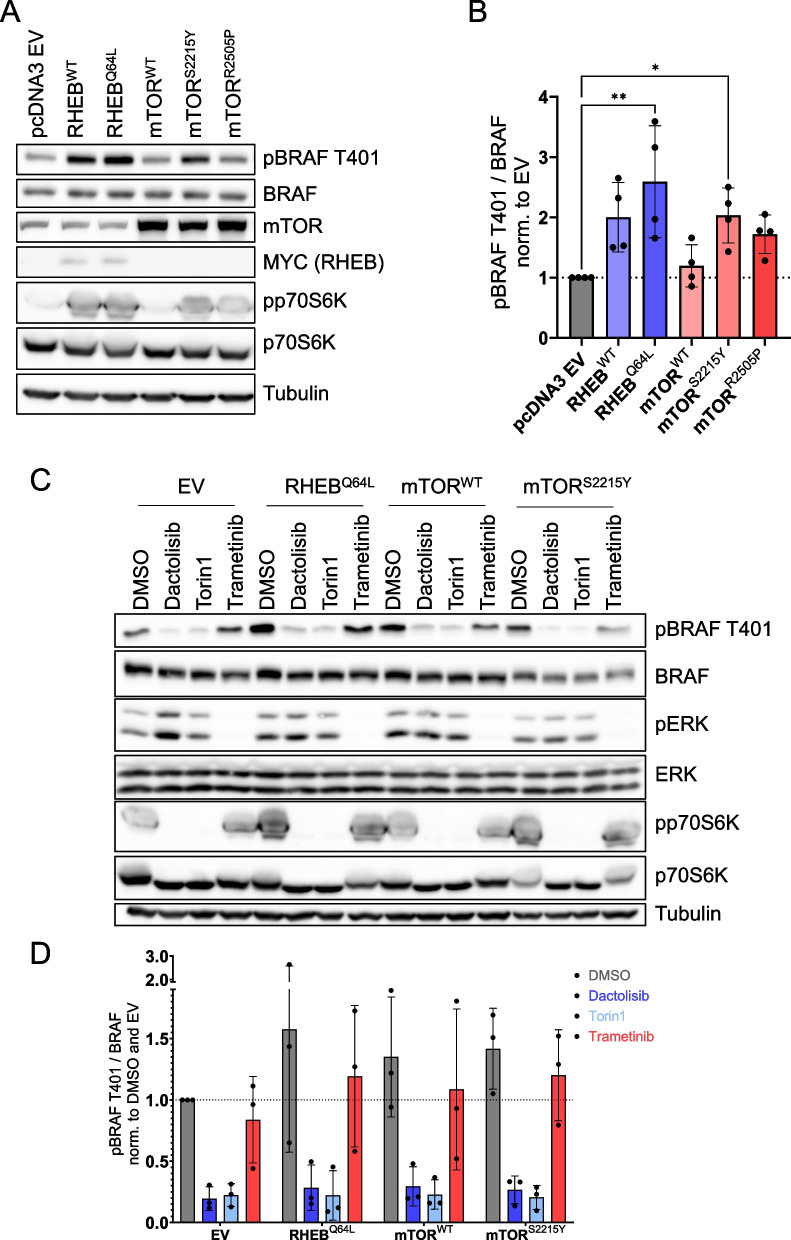
Overexpression of oncogenic RHEB and mTOR mutants increases BRAF phosphorylation at T401, a process reversed by mTOR inhibitors. **A** Representative Western Blot analysis of HEK293T cells ectopically expressing the indicated wildtype (WT) or mutant myc-tagged RHEB or untagged mTOR proteins. Cells transfected with the empty vector (EV) pCDNA3.1 serve as negative control. **B** Quantification of phospho-BRAF T401 per total BRAF (*n* = 4). **C** HEK293T cells were transfected with expression vectors for the indicated RHEB or mTOR proteins, or EV. Forty-eight hours post transfection, cells were treated with the indicated inhibitors for 4 h, lysed and subjected to Western blot analysis. Images represent three independent experiments. **D** Following quantification of Western blot signals for pT401 and total BRAF using FusionCapt Advance Software and normalization to α-Tubulin, the ratio for pBRAF/BRAF was calculated. Ratios were normalized to EV and DMSO. Statistical analysis: mean ± SD, *n* = 3 (**D**) or 4 (**B**), one-way ANOVA with Dunnett ‘s test for multiple comparisons, * *P* ≤ 0.05, ** *P* ≤ 0.01, *** *P* ≤ 0.001, **** *P* ≤ 0.0001. Detection of tubulin and/or total ERK (**C**) serves as loading control

Given the profound effect of mTOR inhibition on pT401, we explored the modulation of the phosphorylation event by extracellular stimuli, as one would expect from a typical MAPK phosphorylation site. First, we assessed whether fetal calf serum (FCS) reduction, either alone or followed by FCS restimulation, influences pT401. As shown in Supplementary Figure S4A, however, these treatments barely modulated pT401, despite a clear induction of ERK phosphorylation upon FCS restimulation. Likewise, a high EGF dose failed to elevate pT401 levels, which were again suppressed by torin1 but not trametinib, despite the latter completely inhibiting ERK phosphorylation (Supplementary Fig. S4B). Collectively, these findings indicate that pT401 exhibits a less dynamic behaviour than previously assumed and is predominantly regulated by mTOR. To further investigate this concept, we hypothesized that genetic activation of the mTOR pathway would increase pT401. First, we compared pT401 levels and mTOR pathway activation in wildtype and constitutive *Tsc1* knock-out MEFs [36]. However, we did not observe detectable alterations of pT401 in the absence of this tumour suppressor, likely due to adaptation phenomena. Nevertheless, dactolisib suppressed pT401 in these two independently generated MEF lines, while trametinib remained ineffective (Supplementary Figure S5). Subsequently, we took the opposite approach and expressed either wildtype or oncogenic mutants of RHEB [37] or mTOR [38] in HEK293T cells. As anticipated, the activating mutants RHEB^Q64L^ and mTOR^S2215Y^ significantly increased pT401 levels (Fig. 3A and B). Additionally, wildtype RHEB, wildtype mTOR, and another tumour-associated mutant, mTOR^R2505P^ induced pT401, albeit to a lesser extent. Importantly, upregulation of pT401 by active RHEB or mTOR proteins was reversed by dactolisib and torin1 but not trametinib (Fig. 3 C and D). Thus, oncogenic activation of the mTOR axis by gain-of-function mutations in two of its tiers increases pT401.

### RPTOR but not RICTOR knockdown cooperates with mTOR inhibition in reducing BRAF phosphorylation at T401

**Fig. 4:**
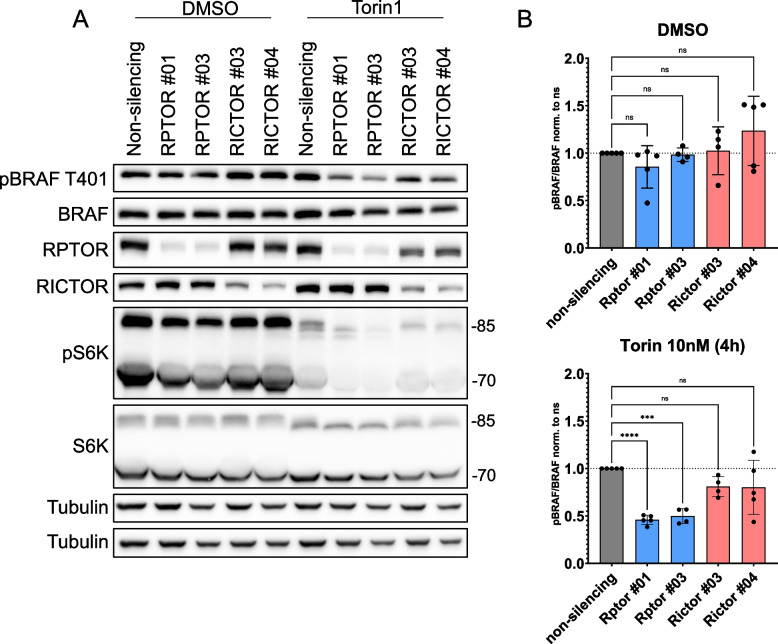
Raptor knock-down sensitizes HEK293T cells to torin1 treatment. Western blot analysis of HEK293T cells stably transduced with indicated dox-inducible pTRIPZ shRNAmir constructs. Following dox treatment for 72 h, cells were either treated with 10 nM torin1 for 4 h or DMSO control. **A** Representative Western blot using the indicated antibodies. **B** Quantification of Western blots normalized either to non-silencing control incubated with DMSO or non-silencing control treated with 10 nM torin1. Following quantification of phospho-BRAF T401 and total BRAF signals using FusionCapt Advance Software and normalization to α-Tubulin, the ratio for pBRAF/BRAF was calculated. Statistical analysis: mean ± SD, *n* = 4 or 5, one-way ANOVA with Dunnett ‘s test for multiple comparisons, * *P* ≤ 0.05, ** *P* ≤ 0.01, *** *P* ≤ 0.001, **** *P* ≤ 0.0001. Detection of tubulin serves as a loading control

The catalytic mTOR subunit occurs in two multi-protein complexes with distinct compositions: mTORC1 and mTORC2, containing Raptor (RPTOR) and Rictor, respectively [11]. Previous findings suggested that BRAF co-purifies with both Rictor from a murine T cell line [39] and with Raptor in MEFs and Caco2 colorectal adenocarcinoma cells [12, 40]. Here, we extend these findings to HEK293T cells (Supplementary Fig. S6A). To assess the relative contribution of both mTOR complexes to pT401, Raptor or Rictor was depleted by doxycycline (dox)-inducible shRNAmirs. Although the turboRFP-coupled shRNAmir cassettes displayed some leakiness in the absence of dox, more than 95% of cells expressed turboRFP following dox treatment (Supplementary Fig. S6B). The homogenous induction of the knockdown cassette was reflected by the reduction of Raptor and Rictor proteins (Fig. 4A). Nevertheless, we did not observe significant differences in BRAF pT401 between the non-silencing control and either Raptor or Rictor targeting shRNAmirs. Upon treatment with a low dose (10 nM) of torin1, however, cells expressing two distinct Raptor targeting shRNAmirs displayed a significant reduction in pT401, compared to cells expressing non-silencing control and Rictor specific shRNAmirs (Fig. 4A and B). The low torin1 dose only had a minor effect on the non-silencing control cell line, as expected (compare to Supplementary Fig. S2A, in which 500 nM of torin1 induced a pronounced reduction of pT401). These findings suggest mTORC1 as the more relevant complex for T401 phosphorylation.

Lastly, we applied a genetic approach to test whether the BRAF^T401A^ mutant affects mTOR signalling. To this end, we transfected HEK293T with either an empty control plasmid or expression vectors for HA-tagged wildtype BRAF (BRAF^WT^) and BRAF^T401A^. In two independent experiments, however, phosphorylation of the mTOR catalytic subunit at S2448, a posttranslational modification correlating with mTORC1 activity [41], did not differ between BRAF^WT^ and BRAF^T401A^ (Supplementary Figure S7). Likewise, we were unable to discern an obvious effect of BRAF^T401A^ on the phosphorylation of the direct mTOR substrate, T389 of pS6K [11]. This suggests that, at least under these experimental conditions, BRAF^T401A^ does not increase mTOR signalling output.

## Discussion

According to the phosphosite database [42], BRAF contains almost 50 phosphorylation sites. Nonetheless, very little is known about the kinases and phosphatases controlling BRAF phosphorylation. Various laboratories identified ERK as the kinase phosphorylating BRAF as part of a negative feedback loop, involving residues S151 and T401, in addition to the C-terminal S^750^PKT^753^P-motif (see Ref. [34] and references therein). Evidence for S151 and T401 as ERK substrates primarily stems from experiments in which NIH3T3 cells were metabolically labelled with ^32^P-orthophosphate followed by stimulation with PDGF [5]. In these convincing experiments, a clear induction of de novo phosphorylation at S151 and T401 was observed. This experimental set-up, however, does not inform about the presence of "cold” phosphate residues already occupying phosphoacceptor sites in BRAF under basal conditions. Indeed, using a highly specific anti-pT401 antibody and MS experiments, we observed prominent pT401 in cells with low or even absent ERK activity ([12], and this study), suggesting that this PTM is less reliant on this pathway than previously thought. Moreover, pT401 was detected in normal breast tissue samples and more than 100 mammary tumours by mass spectrometry [43], further supporting a relatively high abundance of this PTM without acute growth factor stimulation. Additionally, neither naporafenib nor trametinib, despite their ability to confer potent ERK inhibition, reduced pT401 across multiple cell types in our experiments. A very recent study analysing the phosphoproteome of six human pancreatic cancer cell lines using the ERK1/2-selective inhibitor SCH772984 at two different time points did not reveal an obvious regulation of pT401 by ERK [44]. Taken together, all these studies suggest that ERK is not the only and maybe not even the dominant kinase phosphorylating T401. Instead, we provide several lines of evidence supporting T401 as a target of the mTOR pathway. First, structurally and mechanistically distinct mTOR inhibitors, in particular the ATP-competitive compounds torin1 and dactolisib, and to a lesser extent the macrolide antibiotic rapamycin, quench pT401 levels of endogenous BRAF across distinct human cell lines derived from different tissues-of-origin and three independently generated MEF pools. Second, oncogenic RHEB and mTOR mutants increase pT401, which can be acutely reversed by dactolisib or torin1 but not trametinib. While our data do not rule out a role for ERK in pT401 under specific conditions, they strongly implicate the mTOR pathway and maybe mTOR itself as a novel T401 kinase. Indeed, the amino acid residue landscape surrounding T401 matches not only to proline-directed kinases like ERK but also to mTOR [11]. However, we were unable to detect a direct phosphorylation of T401 in in vitro kinase assays using the recombinant catalytic subunit of mTOR and anti-HA purified kinase-dead BRAF^D594A^ as substrate (data not shown). Although this data does not support a kinase-substrate relationship between mTOR and BRAF, it could reflect the necessity of non-catalytic components of the mTOR complexes for the recruitment of BRAF as a substrate. Indeed, Raptor and Rictor, along with mTOR, were detected in BRAF immunoprecipitates from various cell types [12, 39, 45], suggesting that these in vitro kinase assays should be repeated with mTORC1 and 2 holocomplexes, in particular as substrate specificity of mTOR complexes is determined by their composition [11].

Alternatively, the inability of the catalytic mTOR subunit to phosphorylate T401 in vitro could point to a kinase downstream of mTOR or a scenario in which an mTOR-repressed phosphatase targeting pT401 becomes active or increasingly expressed upon mTOR inhibition. For instance, the serine/threonine protein phosphatase 2 (PP2A) interacts with BRAF [12, 40, 46], regulates pT401 status [47] and increases its activity upon mTOR inhibition [48–51]. PP2A also interacts with TSC2 and RHEB [52]. Likewise, several phosphatase components are transcriptionally regulated by torin1 [53]. Calcineurin represents another candidate for an mTOR-regulated phosphatase as it directly dephosphorylates BRAF T401 [31] and inhibits mTOR by dephosphorylation [54, 55]. It is therefore tempting to speculate that mTOR could supress calcineurin activity by a double-negative feedback loop creating bistability until pharmacological disruption. Indeed, tacrolimus, a calcineurin inhibitor, augmented pT401 (Supplementary figure S2F/G), supporting such an interplay between BRAF, mTOR, and calcineurin.

Given the phosphorylation of BRAF by the mTOR pathway, one could imagine a reciprocal relationship between both kinases. Therefore, we investigated whether the BRAF^T401A^ mutant, which can longer be phosphorylated by the mTOR pathway, at least at T401, would affect the mTOR axis. However, we did not detect an obvious effect of BRAF^T401A^ compared to BRAF^WT^ in HEK293T cells. Nevertheless, we surveyed the literature for evidence of mTOR pathway regulation by BRAF. While we could not retrieve any data for physiological conditions, we found three publications showing that BRAF oncoproteins increase mTOR activity. First, Romeo et al. described that expression of oncogenic RAS and BRAF^V600E^ contributes to the constitutive mTOR activity in human melanoma cell lines, an effect mediated in part by the ERK/RSK axis [56]. Second, Faustino et al. [57] provided evidence for a mechanism in which BRAF^V600E^ increases mTOR activity by phosphorylating S428 of LKB1, a kinase which suppresses mTOR via AMPK [57]. Third, Kaul et al. [58] observed that the signature low-grade glioma oncoprotein, the KIAA1549::BRAF fusion, reactivates RHEB by ERK mediated phosphorylation of TSC1/2 and thereby elevates mTOR signaling [58]. Interestingly, this regulation appears cell-type specific, as it only operates in neural stem cells but not in astrocytes [58]. Thus, BRAF might not regulate the mTOR/S6K axis in HEK293T cells. Alternatively, the compared to BRAF^V600E^ and KIAA1549::BRAF much lesser active BRAF^T401A^ mutant might not exceed a critical threshold of ERK signalling required to relieve mTOR suppression by LKB1/AMPK or TSC1/2. Addressing the intricacies of the emerging crosstalk between BRAF and mTOR represents an interesting area for future studies.

## Conclusion

In summary, modulation of the mTOR pathway has a profound effect on the phosphorylation site T401, a residue implicated in the control of BRAF dimerization and stability [5, 14]. Although mechanistic details still await identification, these novel effects of mTOR inhibitors, extensively used in basic research and clinical trials, will stimulate further investigation into the crosstalk between two central axes in oncogenic signalling, the mTOR and ERK pathways.

### Supplementary Information

Additional file 1:  Figure S1. The monoclonal anti-pT401 antibody does not detect the BRAF T401A mutant. Western blot analysis of lysed Plat-E cells overexpressing HA-tagged BRAF WT or the T401A mutant, incubated with 1 µM torin1 or vehicle control for 4 h. Signals for pBRAF T401 and HA are shown. Figure S2. T401 phosphorylation inhibition by torin1 is concentration- and time-dependent, yet more stable than p70S6K phosphorylation. (**A**) Western blot analyses of a single titration experiment showing the concentration-dependent reduction of pT401 following torin1 treatment for 4 h. (**B**) Time course experiment demonstrating the dynamics of T401 phosphorylation loss upon torin1 treatment (1 µM). (**C** and **D**) Quantification of T401 phosphorylation intensities, normalized to total BRAF and a loading control (α-Tubulin) and related to the signal detected in the DMSO control. (**E**) T401 and p70S6K phosphorylation recover following torin1 washout. Western blot analysis of HEK293T cells inhibited with 1 µM torin1 for 4 h, then washed twice with ice-cold PBS, and incubated for the indicated time without an inhibitor prior to lysis. (**F**) Western blot analysis comparing the effects of different mTOR inhibitors, as well as afatinib and tacrolimus, on T401 phosphorylation. HEK293T cells were inhibited for 4 h before lysis and subjected to SDS-PAGE and Western blotting. Detection of tubulin (and vinculin) serves as loading control. Red colour indicates oversaturation of the imager. Figure S3. Quantification of BRAF phosphorylation sites using phosphoproteomics. Quantification of a SILAC-based mass spectrometry experiment. *Braf*^−/−^ murine embryonic fibroblasts (MEFs) expressing ER T2 -HRAS G12V were transduced with human HA-tagged BRAF and treated for 24 h with either 4HT or ethanol (solvent control). Subsequently, cells were either incubated with torin1 or DMSO (vehicle) 4 h prior to lysis. Then, HA-tagged BRAF was immunoprecipitated, digested, and the abundance of the indicated phosphorylation sites was analysed. Shown is the fold change of one biological replicate of the phosphorylation of the indicated site of ER T2 -HRAS^G12V^ on vs. ER T2 -HRAS^G12V^ off (**A**) and of torin1/DMSO in the presence of ER T2-HRAS^G12V^ switched on (**B**). Error bars show the coefficient of variability over all redundant quantifiable peptides. (**C**) T401 peptide normalised occupancy was calculated for the three conditions as published previously [35]. Figure S4. BRAF T401 phosphorylation is neither enhanced by fetal calf serum nor EGF. (**A**) Western blot analysis of HEK293T cells that were incubated in medium containing the indicated percentage of FCS for 18 h. Samples in lanes 5 and 6 were derived from cells incubated in 1% FCS for 17 h followed by restimulation a higher FCS concentration (10% or 5%) for one hour. (**B**) HEK293T cells were pre-treated with either torin1 (500 nM), trametinib (Tram, 100 nM), or vehicle control for 4 h and then either left untreated or treated with hEGF (100 ng/mL) for 15 min. Detection of tubulin serves as a loading control. Figure S5. Constitutive Tsc1 deficiency does not affect BRAF T401 phosphorylation. Western blot analysis of TSC1 knock-out and control MEFs treated with either dactolisib (Da, 1 µM), trametinib (T, 100 nM) or DMSO (**D**) as a control for 4 h. Detection of tubulin serves as a loading control. Figure S6. BRAF co-purifies with Raptor and Rictor. (**A**) Co-immunoprecipitation of endogenous Raptor and Rictor with purified HA-tagged BRAF WT . (**B**) FACS Analysis of the generated Raptor and Rictor knock-down HEK293T cell lines. Cells were treated with 500 ng/mL doxycycline for 48 h before being subjected to FACS analysis. shRNA constructs additionally encode turboRFP to distinguish cells expressing the transduced shRNA constructs. Figure S7. BRAF T401A does not change mTORC1 signalling. Western Blot of HEK293T cells transfected with either pMIberry empty vector, HA-tagged BRAF wild-type or the BRAF T401A mutant. Lysates were prepared 48 h after transfection. Two independent biological replicates were used and membranes were probed with indicated antibodies. For the left replicate Tubulin, and for the right panel Vinculin served as loading control.

## Data Availability

No datasets were generated or analysed during the current study.

## Availability of data and materials

All data generated or analysed during this study are included in this published article [and its supplementary information files].
