# Supplementary material for: The mTOR pathway controls phosphorylation of BRAF at T401
Source: Cell Commun Signal. 2024 Sep 2;22:428. doi: 10.1186/s12964-024-01808-2 (PMC11370054; doi:10.1186/s12964-024-01808-2)

Supplementary Figure S1

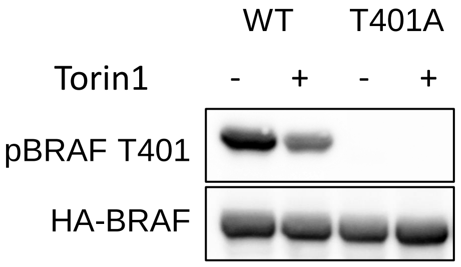

Supplementary Figure S2

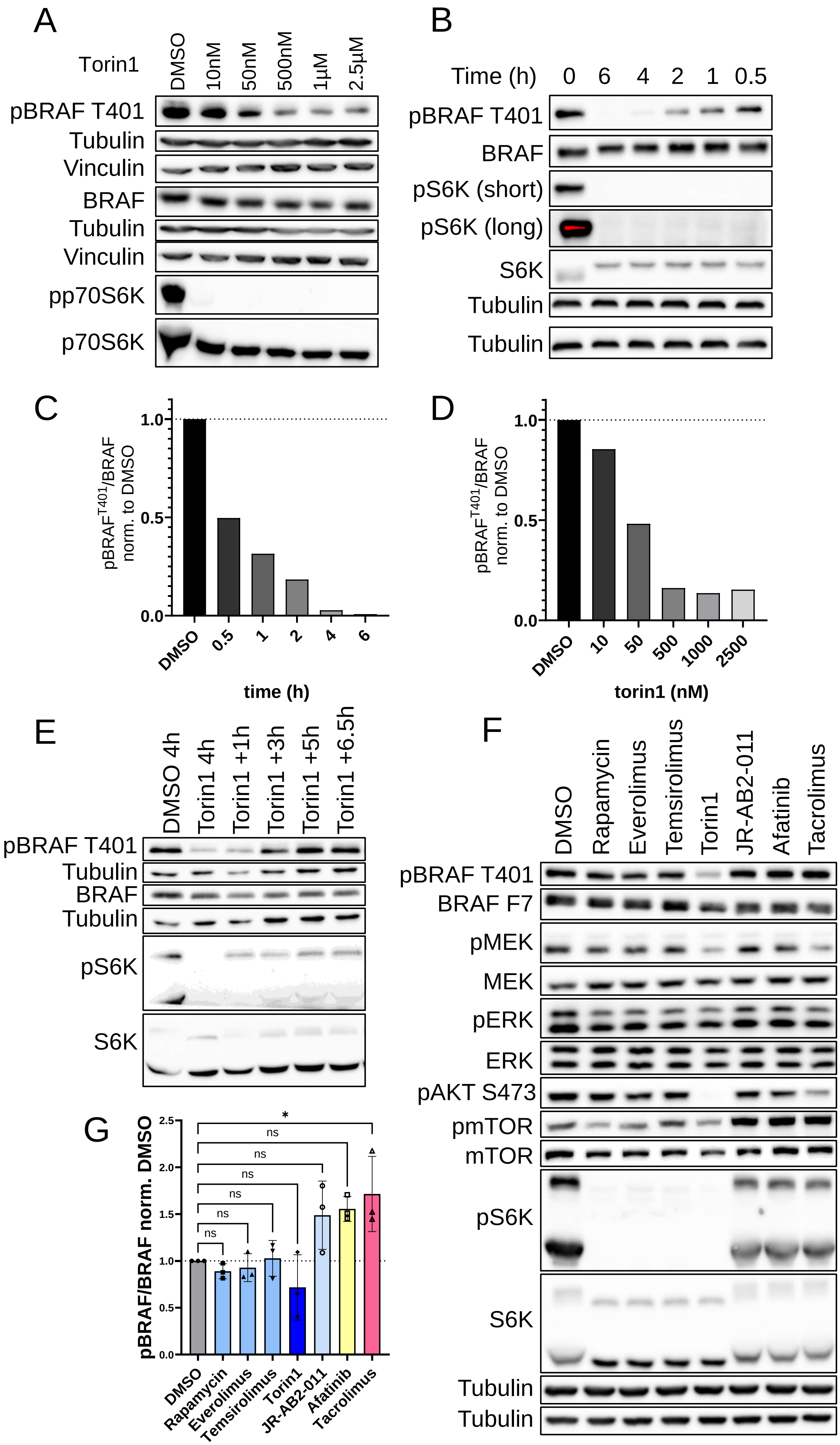

Supplementary Figure S3

A

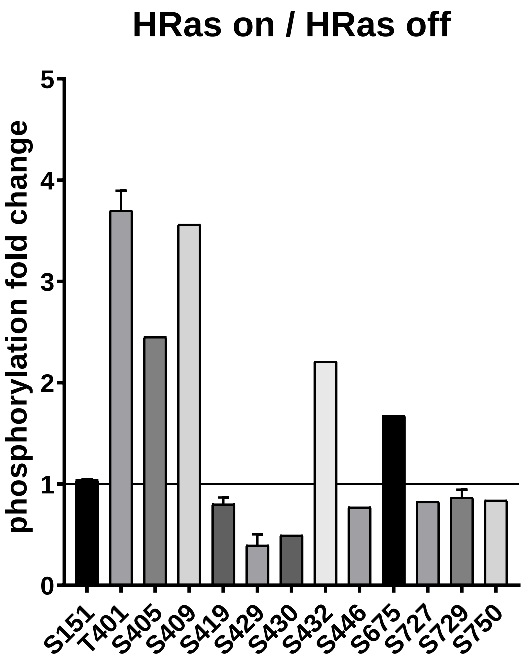

B

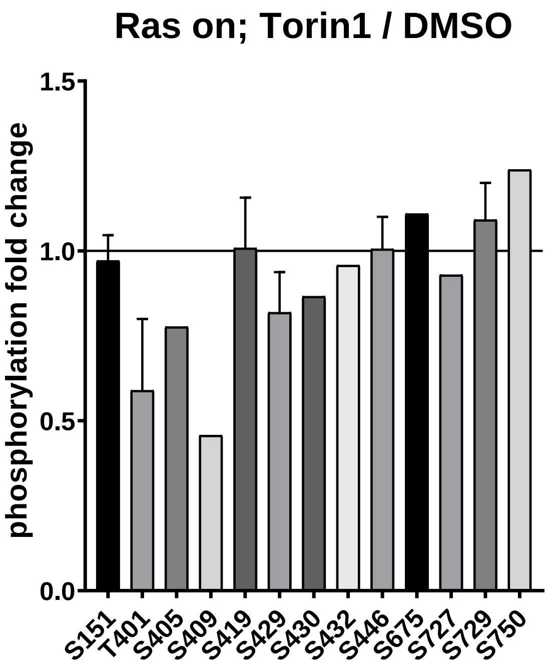

C

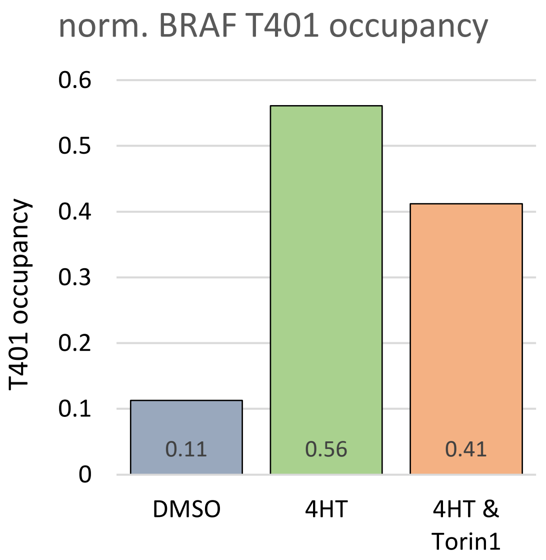

Supplementary Figure S4

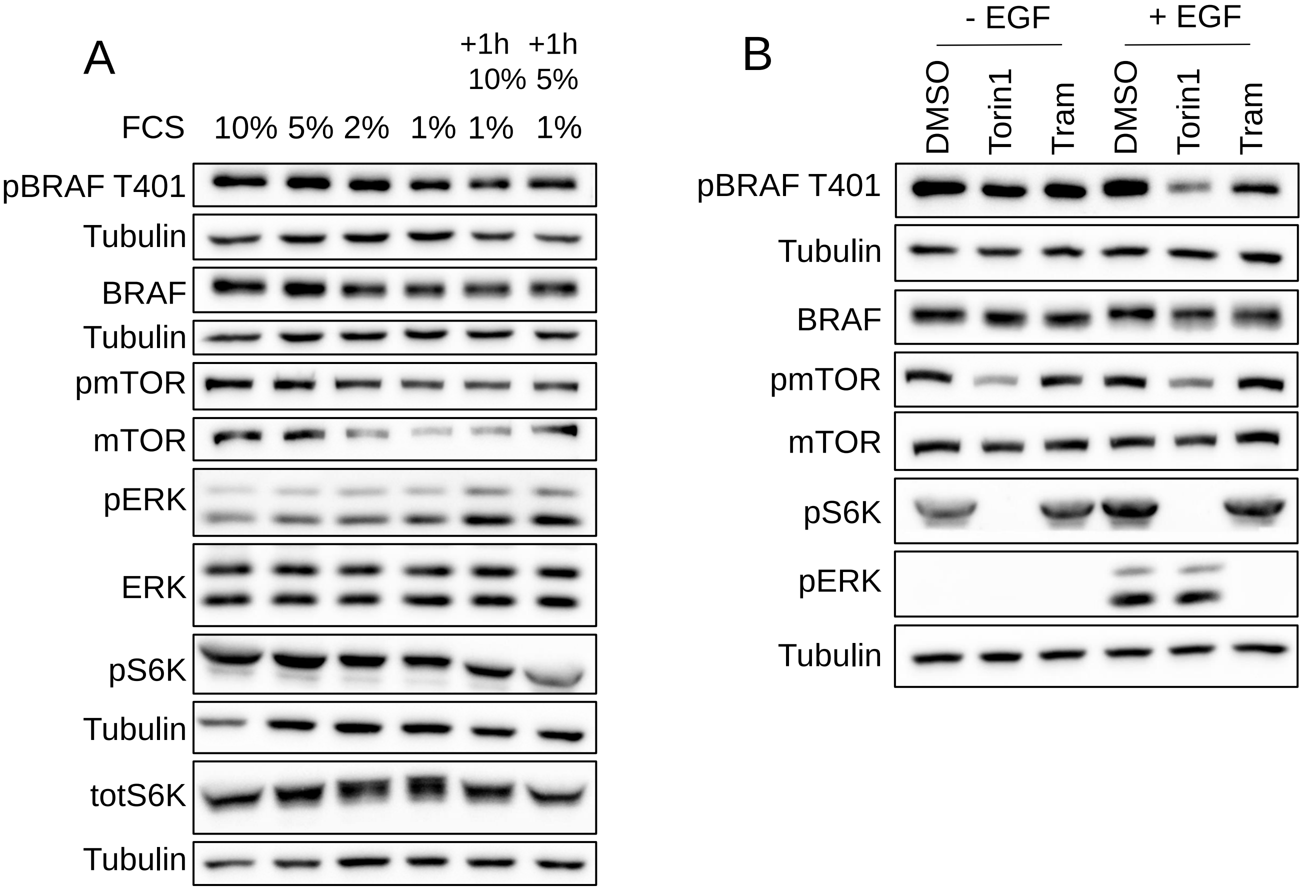

Supplementary Figure S5

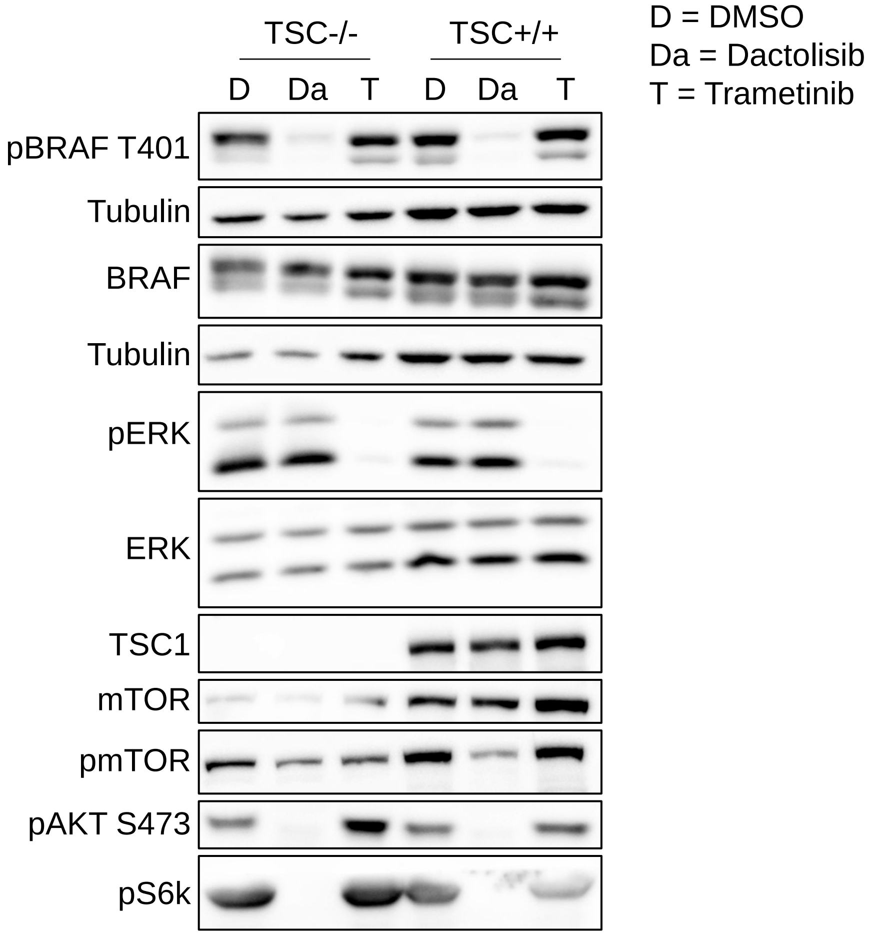

MEFs

Supplementary Figure S6

A

CO-IP of BRAF^{WT}: RPTOR and RICTOR

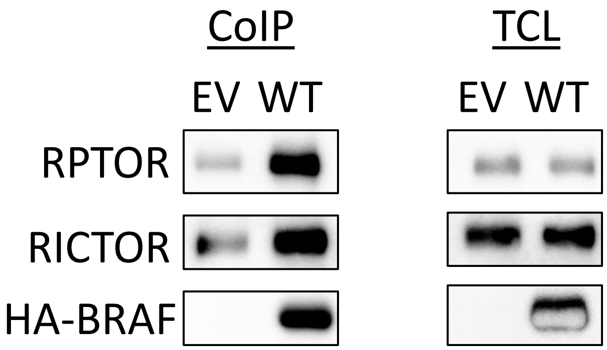

B

FACS analysis of Rptor & Rictor knock-down cell lines

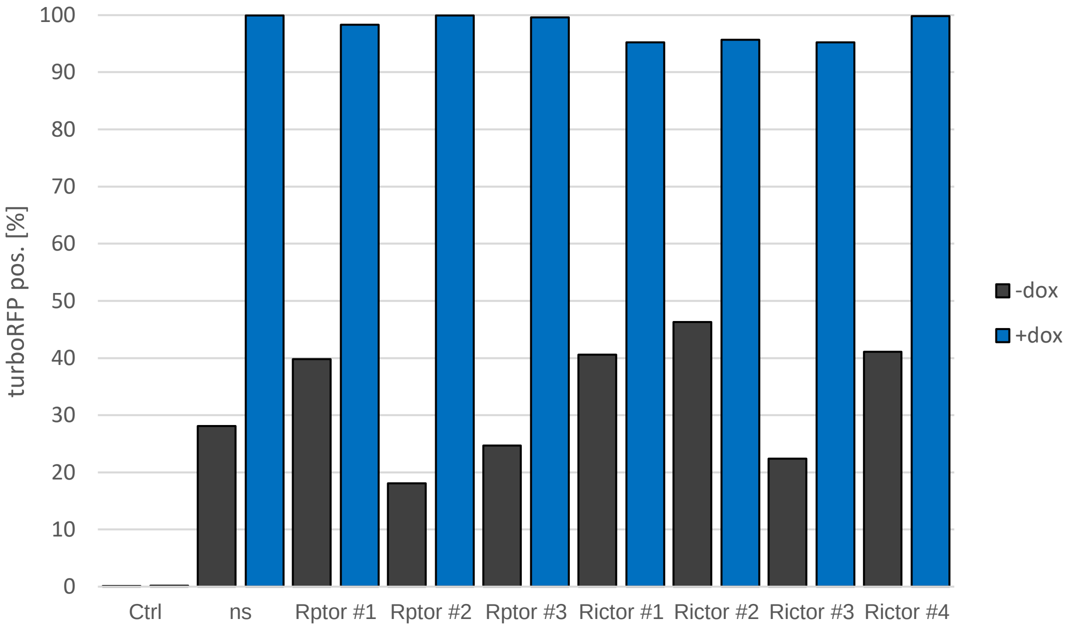

Supplementary Figure S7

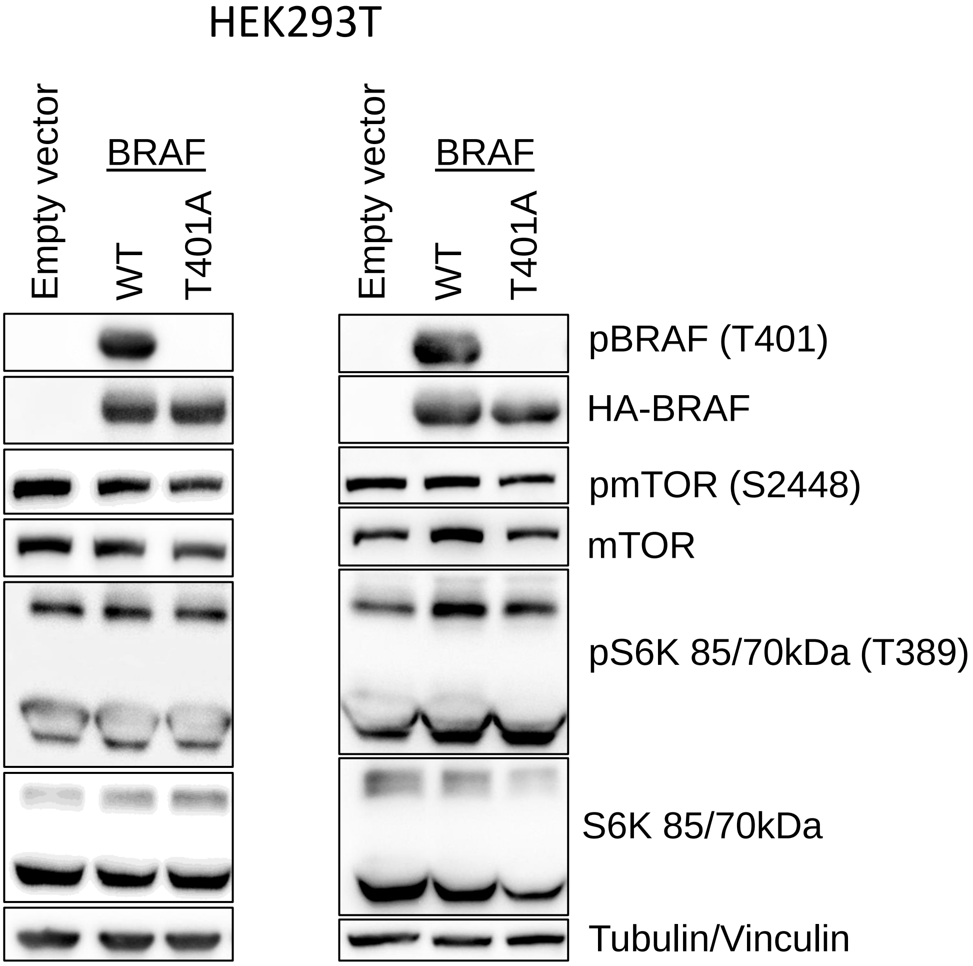

Supplement: Supplementary file 1 — Additional file 1: Figure S1. The monoclonal anti-pT401 antibody does not detect the BRAF T401A mutant. Western blot analysis of lysed Plat-E cells overexpressing HA-tagged BRAF WT or the T401A mutant, incubated with 1 µM torin1 or vehicle control for 4 h. Signals for pBRAF T401 and HA are shown. Figure S2. T401 phosphorylation inhibition by torin1 is concentration- and time-dependent, yet more stable than p70S6K phosphorylation. (A) Western blot analyses of a single titration experiment showing the concentration-dependent reduction of pT401 following torin1 treatment for 4 h. (B) Time course experiment demonstrating the dynamics of T401 phosphorylation loss upon torin1 treatment (1 µM). (C and D) Quantification of T401 phosphorylation intensities, normalized to total BRAF and a loading control (α-Tubulin) and related to the signal detected in the DMSO control. (E) T401 and p70S6K phosphorylation recover following torin1 washout. Western blot analysis of HEK293T cells inhibited with 1 µM torin1 for 4 h, then washed twice with ice-cold PBS, and incubated for the indicated time without an inhibitor prior to lysis. (F) Western blot analysis comparing the effects of different mTOR inhibitors, as well as afatinib and tacrolimus, on T401 phosphorylation. HEK293T cells were inhibited for 4 h before lysis and subjected to SDS-PAGE and Western blotting. Detection of tubulin (and vinculin) serves as loading control. Red colour indicates oversaturation of the imager. Figure S3. Quantification of BRAF phosphorylation sites using phosphoproteomics. Quantification of a SILAC-based mass spectrometry experiment. Braf−/− murine embryonic fibroblasts (MEFs) expressing ER T2 -HRAS G12V were transduced with human HA-tagged BRAF and treated for 24 h with either 4HT or ethanol (solvent control). Subsequently, cells were either incubated with torin1 or DMSO (vehicle) 4 h prior to lysis. Then, HA-tagged BRAF was immunoprecipitated, digested, and the abundance of the indicated phosphorylati [file 12964_2024_1808_MOESM1_ESM.pdf]
